# Possible link between steatotic liver diseases, severe COVID-19 and cognitive impairment in post-COVID-19 syndrome

**DOI:** 10.1007/s15010-025-02531-x

**Published:** 2025-04-10

**Authors:** Philipp A. Reuken, Freya Wagner, Kathrin Finke, Christina Lemhöfer, Christian Puta, Sven Stengel, André Scherag, Jan-Christoph Lewejohann, Andreas Stallmach, Stefanie Quickert

**Affiliations:** 1 Department of Internal Medicine IV (Gastroenterology, Hepatology, and Infectious Diseases), Jena University Hospital, Am Klinikum 1, 07747 Jena, Germany; 2 Department of Neurology, Jena University Hospital, Jena, Germany; 3 Institute of Physical and Rehabilitation Medicine, Jena University Hospital, Jena, Germany; 4 Department of Sports Medicine and Health Promotion, Friedrich-Schiller-University Jena, Jena, Germany; 5 Department of Neuropediatrics, Jena University Hospital, Jena, Germany; 6 Institute of Medical Statistics, Computer and Data Sciences, Jena University Hospital, Jena, Germany; 7 Department of Emergency Medicine, Jena University Hospital, Jena, Germany; 8 Center for Sepsis Control and Care (CSCC), Jena University Hospital, Jena, Germany; 9 Interdisciplinary Centre for Clinical Research (IZKF) Jena, Jena University Hospital, Jena, Germany

**Keywords:** Post-COVID, Fatigue, Cognitive dysfunction, SLD, Hepatic steatosis

## Abstract

**Purpose:**

Steatotic liver diseases (SLD) have become more prevalent over the last decade and are associated not only with cardiometabolic diseases but also with psychological symptoms (depression, fatigue). These symptoms are also common in post-COVID syndrome (PCS). Therefore, the aim of the study was to analyze the burden of SLD in PCS patients.

**Methods:**

We systematically screened all PCS patients from our post-COVID outpatient clinic using transient elastography, structured questionnaires for neurocognitive evaluation and blood sample analysis. Controls without PCS and without known liver diseases were also recruited and assessed with the same approach.

**Results:**

560 PCS patients and 103 healthy controls were included. The overall prevalence of SLD was high in both cohorts (57 vs. 53%). PCS patients with SLD were more frequently male (41 vs. 24%), older (52 vs. 44 years) and had more cardiometabolic diseases (87.0 vs. 46.4%). Cognitive impairment was more related to SLD in PCS patients than in the no-SLD group (OR: 1.68, CI: 1.14–2.46, p = 0.008). The presence of SLD was related to severe COVID-19 with hospitalization (OR: 2.91, CI: 1.85–4.56, p < 0.001). Within 1 year of the follow-up, 152 of 289 patients described a resolution in PCS irrespective of the presence or absence of SLD (log-rank p = 0.96).

**Conclusions:**

SLD is associated with severe COVID-19 and cognitive dysfunction in PCS. Longitudinal studies are needed to assess the role of hepatic steatosis, development of post-acute infection regulation (e.g., SARS-CoV-2) and to differentiate between SLD-associated symptoms and PCS.

**Supplementary Information:**

The online version contains supplementary material available at 10.1007/s15010-025-02531-x.

## Main text

### Introduction

Shortly after occurrence of the first cases of SARS-CoV-2 infection, it became evident, that a significant proportion of patients suffered from long lasting symptoms. Approximately 0.7–1.4% of infected patients developed long-lasting symptoms, depending on the variant they were infected with and on previous vaccinations [1, 2]. These symptoms are defined as post-COVID syndrome (PCS) when lasting for 12 weeks or longer without another explanation and have impact on quality of life [3].

Until today, more than 200 different symptoms have been associated with PCS [4, 5] with fatigue including ME/CFS and cognitive dysfunction emerging as the most common [6–9]. To evaluate functional status as well memory impairments in these patients structured questionnaires are often used in healthcare and in studies: for mental health the Global Assessment of Functioning score (GAF), for fatigue the Brief Fatigue Inventory (BFI) and the Fatigue Assessment Scale (FAS), for depression the Patient Health Questionnaire (PHQ-9) and for cognitive dysfunction the Montreal Cognitive Assessment (MoCA) [9].

Although the exact pathophysiology underlying PCS is still unknown, discussed pathways include microvascular alterations and immunometabolic interactions [10] autoimmunology, chronic inflammation, viral persistence, direct organ damage, endothelial dysfunction, or metabolic changes [11]. Female sex, older age, and higher BMI were associated with an increased risk of developing PCS in a recent meta-analysis [12]. Additionally, comorbidities (anxiety, depression, asthma, chronic kidney disease, chronic obstructive pulmonary disease, diabetes mellitus, immunosuppression, and ischemic heart disease) were identified as independent risk factor (OR = 2.48; 95% CI, 1.97–3.13) [12]. Although this meta-analysis did not report on liver diseases as comorbidities, a recent study reported that metabolic dysfunction-associated steatotic liver disease (MASLD) was associated with PCS in a follow-up of a cohort of patients who were hospitalized for treatment of a severe acute infection [13].

In the acute phase of the infection elevated liver enzyme levels reflecting hepatic injury are common in COVID-19 patients with and without chronic liver diseases [14], especially in male, elderly and overweight patients [15]. Literature remains elusive whether liver dysfunction remains in PCS or might lead to changes in liver architecture. Moreover, in acute SARS-CoV-2 infections, chronic liver diseases contribute to increased mortality rates [16]. Using a precision PCS phenotyping algorithm, one retrospective case–control study [17] showed that the gastrointestinal category contains 11% of patients with liver associated symptoms. It is remarkable that chronic liver diseases are associated with fatigue not only in cases of SLD but also in other liver disease, such as primary biliary cholangitis [18]. This finding and the previously mentioned association of MASLD as one important form of SLD and PCS represent a potentially important link given the high number of (MA)SLD patients worldwide [19]. The prevalence of SLD is estimated at about 42–44% and is primarily attributed to lifestyle factors, e.g. obesity, high calorie intake, low physical activity and alcohol [20–22].

It should also be noted that the definition of SLD has recently changed according to the Delphi Consensus. While non-alcoholic fatty liver diseases were previously considered a diagnosis of exclusion, the most recent definition includes several subcategories of SLD. The most common is MASLD, which applies to patients with SLD and cardiometabolic risk factors. Alcoholic liver disease (ALD) is defined in patients with SLD and increased alcohol consumption, while metALD describes patients who meet MASLD criteria but also have increased alcohol intake [23].

In the context of persistent inflammation, neuroinflammation has emerged as a notable consequence of PCS [10]. It is suggested to result from a combination of airway inflammation and systemic chemokine release. Furthermore, there is acknowledgment of direct organ damage, particularly affecting the brainstem and the hypothalamic-pituitary axis [24].

In SLD, neuroinflammation is evident [25, 26]. Depression, anxiety, and cognitive decline are linked to SLD [27–29]; gray matter abnormalities may link SLD to depression [30]. Screening tests such as the MoCA reveal significant cognitive impairment in SLD patients, including both MASLD [31] and ALD [32] patients.

Therefore, the aim of our study was to evaluate the burden of hepatic steatosis in PCS, independent of the etiology of liver disease and independent of the course of acute SARS-CoV-2 infection.

## Patients and methods

### Study design and patient cohorts

**Fig. 1:**
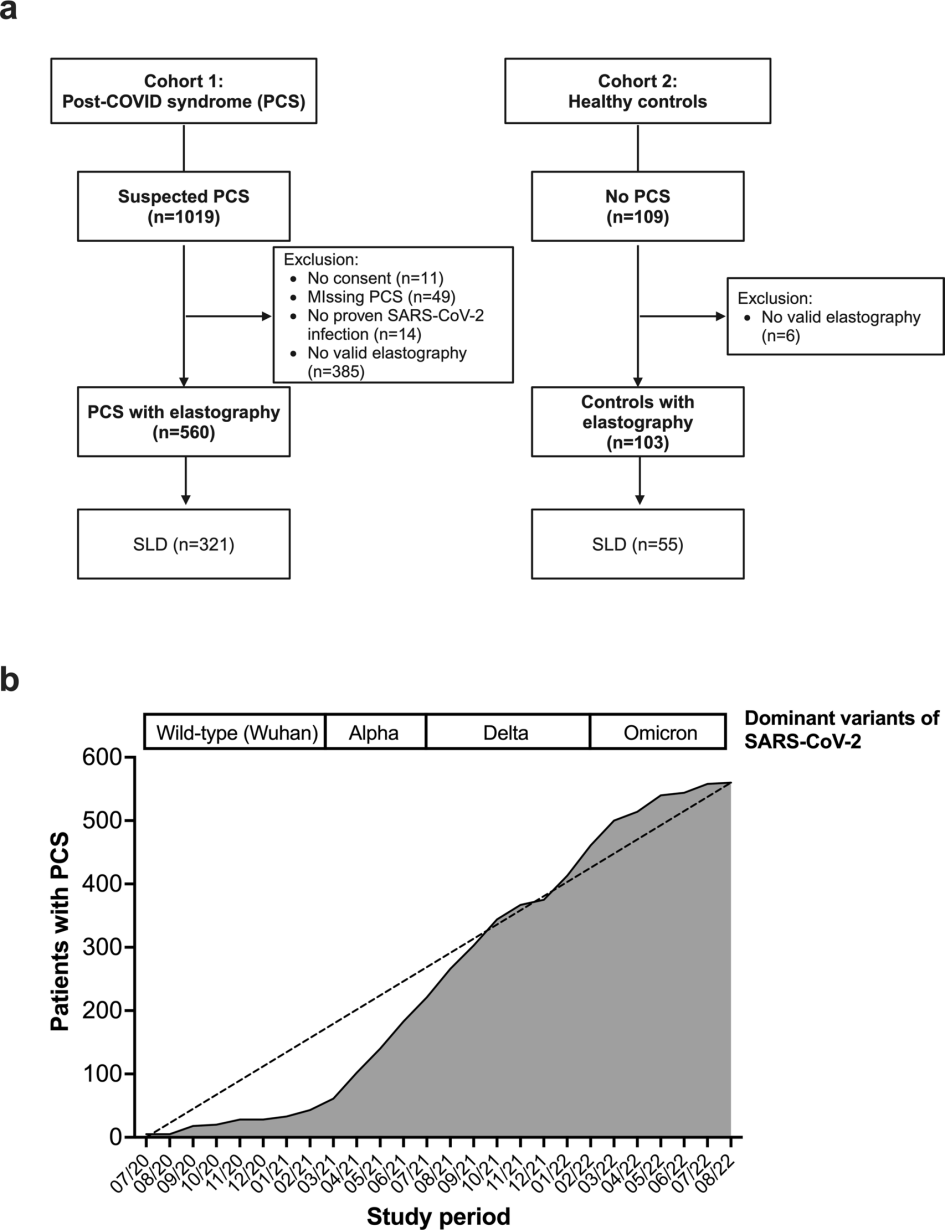
Overview of the study. **A** Flow chart of the two cohorts: patients with post-COVID syndrome (PCS) and controls without PCS, with the proportion with steatotic liver diseases (SLD), including excluded cases. Created with biorender.com. **B** Cumulative recruitment of patients with PCS and valid elastography over the study period (July 2020 und August 2022) according to the dominant variant of SARS-CoV-2

Patients attending at the post-COVID outpatient clinic of the Jena University Hospital (Germany) between July 2020 and August 2022 were enrolled consecutively and examined when they received an ultrasound with transient elastography (TE) as cohort 1, as previously published [6]. Additionally, a comparison cohort of healthy participants without PCS was enrolled between November 2023 and February 2024 (cohort 2). This means that healthy subjects do not attribute any existing symptoms/complaints to a previous SARS-CoV-2 infection, or the controls have not yet had a suspected or proven SARS-CoV-2 infection. The study has been approved by the institutional ethics committee of Friedrich-Schiller-University Jena (cohort 1: 2020–1978-Daten, cohort 2: 2023–2952-BO). **(**Fig. 1**).**

In cohort 1, inclusion criteria comprised: (1) Minimum age of 18 years, (2) confirmed SARS-CoV-2 infection via antigen or PCR test, (3) presence of PCS (defined as self-reporting symptoms persisting for more than 12 weeks after SARS-CoV-2 infection without other explanation according to UK National Institute for Health and Care Excellence (NICE) [33] and (4) valid TE examination. Cohort 2 inclusion criteria were: (1) Age between 40 and 65 years (to achieve almost age-matched cohorts), (2) no PCS and (3) no known chronic liver disease. Data collected from both cohorts included: gender, age, BMI, date of positive SARS-CoV-2 test, comorbidities, overall condition during and after the infection as well as any occurring symptoms, hospitalization due to SARS-CoV-2 infection with associated medication, ventilation or imaging, TE examination score, questionnaires on global functional status, fatigue and a neurocognitive screening (GAF score, FAS, BFI, and PHQ-9, as well as MoCA), laboratory analyses as well as severity of SARS-CoV-2 infection. Regarding the definition of the severity of SARS-CoV-2 infection, we employed two distinct scales: Initially, for expeditious sorting of patients during their visit to our outpatient clinic, we utilized a 3-point scale categorizing SARS-CoV-2 infection severity into (1) non-severe (various symptoms of illness without additional need of oxygen supplementation), (2) severe (spO2 < 90–94% on room air, respiratory rate > 30/min, radiological signs of pneumonia) and (3) critical (necessity of mechanical ventilation or other types of organ support) (adapted and modified from the “Epidemiology Working Group for Ncip Epidemic Response and Prevention” [34]). Subsequently, to further delineate infection severity, we introduced a 7-point scale. This extended scale allowed us to classify our patients into the following infection severity categories: (1) not hospitalized, no limitations in activities (2) not hospitalized, limitations in activities (3) hospitalized, not requiring supplemental oxygen, (4) hospitalized, requiring supplemental oxygen, (5) hospitalized, requiring NIV/HFNC, (6) hospitalized, requiring invasive ventilation/ECMO, (7) Death. Exclusively in Cohort 1, we assessed the date of the last visit to the post-COVID outpatient clinic and whether PCS persisted. Moreover, cohort 1 participants attended follow-up visits regularly at intervals of three months to one year. Recovery from PCS was defined as resolution of self-reported symptoms in standardized questionnaires [35] (especially no presence of neurocognitive dysfunctions), which does not require re-admission to the clinic.

### Questionnaires on self-reporting symptoms, functional status and fatigue and neurocognitive screening

Participants completed prospective questionnaires on current self-reporting symptoms, the GAF, the depression module of the PHQ-9 [36], the FAS [37] and the BFI [38]. The GAF score ranges from 0 to 100. Participants rated their subjective well-being during their infection and on the day of their visits to our clinic. A maximum score of 100 points indicated complete well-being (described as “holiday feeling”), while a minimum score of 0 reflected severe illness (comparable to an “intensive care unit stay”). The PHQ-9 assess the severity of depression symptoms across nine items. Patients and participants with a score below 10 points were categorized as having no clinically relevant depressive symptoms. Symptoms of depression were divided into mild (10–14 points), intermediate (15–19 points) or severe (20–27 points as a maximum score). The FAS measures severity and impact of fatigue across 10 items encompassing both physical and mental aspects of fatigue, as well as their effect on daily activities. The maximum score of the FAS is 50 points: a score of 22 points or higher indicates signs of above-average fatigue. The BFI focusses on the impact of fatigue on daily functioning as well as mood and assessed with nine items. The maximum score is 90; severity of fatigue is classified as mild (9–35 points), moderate (36–62 points) or severe (63–90 points). The MoCA [39] is a brief screening of cognitive functioning with a maximum score of 30. Values below the cut-off of 26 were considered indicative of cognitive dysfunction, while values above 26 were classified as indicative of normal cognitive function.

### Comorbities and cardiometabolic risk factors

Comorbities were categorized as in the Charlson Comorbidity Index [40]. Overweight and obesity were defined using the body mass index (BMI) with the thresholds ≥ 25 kg/m^2^ and ≥ 30 kg/m^2^, respectively, as recommended by the WHO [41]. Overweight, diabetes mellitus type 2, dyslipidemia (as defined by plasma triglycerides ≥ 1.70 mmol/l or HDL-cholesterol ≤ 1.0 mmol/l) or arterial hypertension (exceeding 130/85 mmHg) were identified as cardiometabolic risk factors according to the Delphi consensus statement on MASLD [23]. We did not use the waist circumference as potential substitute for BMI because it was not routinely measured and patients were enrolled in the PCS cohort between 2020 and 2022, before the new consensus recommendation. Alcohol intake was self-reported by the patients, and for many of them the information is missing.

### Detection of steatotic liver diseases using ultrasound and/or transient elastography

We assessed liver steatosis with ultrasound and TE, a non-invasive ultrasound-based method, using a FibroScan® mini + 430 (Fa Echosens, Bonn, Germany). In the study group (PCS) we performed both methods, but in the controls only the TE. TE measurements were performed with M or XL probes, with the XL probe used for certain, mostly obese, patients as recommended by the machine. A valid TE includes 10 measurements per patient with a median value given. Steatosis was considered likely if the CAP (Controlled Attenuation Parameter) value was above 248 dB/m with the following grading: Steatosis grade S1 (mild) ≥ 248 db/m, S2 (moderate) ≥ 268 db/m and S3 (severe) ≥ 280 db/m [42]. Although there are different CAP cut-offs for MASLD [43], ALD [44] and metALD [45, 46], we did not apply etiology-specific CAP cut-offs due to insufficient data on alcohol consumption and assessment of cardiometabolic risk factors in our cohorts. Significant fibrosis (F2) was considered present in TE at an LSM (Liver Stiffness Measurement) value of ≥ 7 kPa, while advanced fibrosis (F3) and cirrhosis (F4) were defined at thresholds of ≥ 10 kPa and ≥ 13 kPa, respectively [47].

### Statistical analysis

For categorical variables, absolute and relative frequencies are provided, and between-group comparisons were performed using Fisher's exact or Chi-squared tests. For metric variables median with interquartile range (25–75%) are given. Mann–Whitney and Wilcoxon test were applied to unpaired and paired analyses, respectively. In addition, Spearman’s rank correlation was computed to identify the relationship between two metric variables. To identify potential predictors for SLD we performed logistic regression models (univariate). Risk factors for PCS recovery were determined via Cox regression models (both univariate or multivariate). The odds ratios (OR) and 95% confidence intervals were given in all regression analysis. As part of time-to-event analyses, we provide Kaplan–Meier estimates, used log-rank tests for the comparison of the two groups and regression models to determine potential risk factors for a long-lasting post-COVID-19 course.

While the sample size of cohort 1 (PCS group) resulted from the volume of patients in our post-COVID outpatient clinic, the sample size of cohort 2 (healthy control group) was estimated to compare the prevalence of SLD with the PCS cohort. Based on the literature [20–22], a prevalence of SLD of ~ 43% we assumed in the population. To estimate such a rate with an adequate 95% exact Clopper-Pearson confidence interval (in this case ± 10.07%), at least 100 control participants are required. For a sample size of n = 200, n = 300, n = 400, the precision would be ± 7.07%, ± 5.74% and ± 4.96% respectively.

The heatmaps were created using R and heatmap.2 from the gplots package (version 3.2.0). The hierarchical cluster analysis was performed using Euclidean methods for measuring distances and complete methods of the R function hclust.

In general, we applied a two-sided significance level of α = 0.05 for all tests and did not correct of multiple comparison testing, as this is an exploratory analysis. We decided to display *P* values categorized as: * *P* < 0.05, ** *P* < 0.01, ** *P* < 0.001.

In this study, all statistical analyses were realized with SPSS version 29 (IMB, Armonk, NY), Prism 10 (GraphPad, La Jolla, CA) and R software version 4.4.2.

## Results

**Fig. 2:**
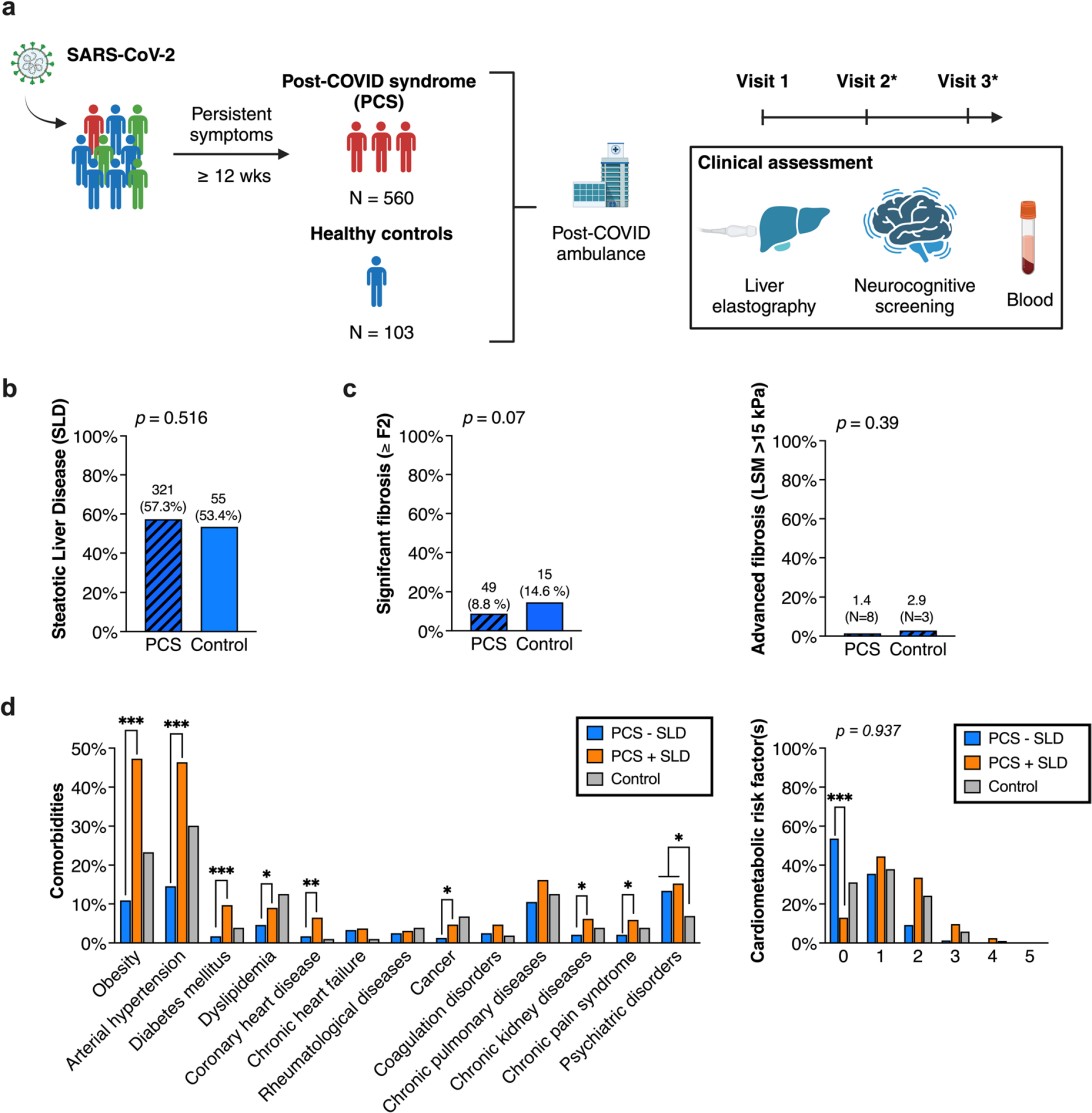
Steatotic liver diseases (SLD) in post-COVID-19 syndrome (PCS). **A** Scheme of the study design. *Visit 2/3 only in PCS patients. Created with biorender.com. **B** Prevalence of SLD, assessed by liver elastography compared between patients with PCS (N = 560) and healthy controls (N = 103). **C** Frequency of significant fibrosis (≥ F2) and advanced fibrosis > 15 kPa in LSM is shown. **D** Presence of comorbidities and cardiometabolic risk factor(s). Statistical test: Fisher’s exact test in B, C and D

We included 560 PCS patients in cohort 1 **(**Fig. 1**)**, who were predominantly female (65.9%) and had a median age of 50 years and a median BMI of 27 kg/m^2^ (IQR 23.5–30.9). Overall, 401 (71.6%) of the patients had at least one cardiometabolic risk factor or comorbidity. Arterial hypertension (32.9%), obesity (31.7%), psychiatric disorders (14.5%), and chronic pulmonary diseases (13.8%) were the most common comorbidities. The majority of the patients had a non-severe course of the acute infection (78.6%), while hospitalization was required in 21.4%. **(**Table 1**, **Fig. 2**).**

**Table 1:** Clinical characteristics of patients with and without steatotic liver diseases (SLD) in post-COVID-19 syndrome and healthy controls without long-term-sequelae after SARS-CoV-2 infection. *P* values from Mann-Whitney, Fisher's exact or Chi-squared test (bold numbers indicate statistical significance)

	Post-COVID-19 patients, n = 560							Healthy controls, n = 103		
	Overall		SLD, n = 321		No-SLD, n = 239		*P* value	Overall		*P* value
	N		N		N			N		
Age (years)	560	50 (39–57)	321	52 (43–59)	239	44 (33–54)	** < 0.001**	103	55 (49–59)	** < 0.001**
Female sex	560	369 (65.9)	321	188 (58.6)	239	181 (75.7)	** < 0.001**	103	61 (59.2)	0.217
BMI (kg/m^2^)	558	27.0 (23.5–30.9)	319	29.7 (26.3–33.0)	239	23.8 (21.6–26.6)	** < 0.001**	103	26.6 (23.2–29.8)	0.244
**Previous SARS-CoV-2 infection**										
Confirmed infection	560	560 (100)	321	321 (100)	239	239 (100)	-	103	90 (87.4)	** < 0.001**
COVID-19 severity (7-point ordinal scale) [61] (1) not hospitalized, no limitations on activities (2) not hospitalized, limitation on activities (3) hospitalized, not requiring supplemental oxygen (4) hospitalized, requiring supplemental oxygen (5) hospitalized, requiring NIV/HFNC (6) hospitalized, requiring invasive ventilation/ECMO	560	9 (1.6) 431 (77.0) 34 (6.1) 46 (8.2) 19 (3.4) 21 (3.8)	321	6 (1.9) 223 (69.5) 22 (6.9) 34 (10.6) 18 (5.6) 18 (5.6)	239	3 (1.3) 208 (87.4) 12 (5.0) 12 (5.0) 1 (0.4) 3 (1.3)	** < 0.001**	90	12 (13.3) 76 (84.4) 0 2 (2.2) 0 0	** < 0.001**
COVID-19 severity (3-point scale, WHO 2023) [62] (1) Non-severe (2) Severe (3) Critical	560	440 (78.6) 80 (14.3) 40 (7.1)	321	251 (78.2) 34 (10.6)) 36 (11.2)	239	211 (88.7) 24 (10.0) 4 (1.7)	** < 0.001**	90	88 (97.8) 2 (2.2) 0	** < 0.001**
Hospitalization	560	120 (21.4)	321	92 (28.7)	239	28 (11.7)	** < 0.001**	90	2 (2.2)	** < 0.001**
ICU stay	559	39 (7.0)	321	34 (10.6)	238	5 (2.1)	** < 0.001**	90	0	**0.004**
COVID-19 pneumonia (in chest imaging: CT/X-ray)	560	90 (16.1)	321	72 (22.4)	239	18 (7.5)	** < 0.001**	90	2 (2.2)	** < 0.001**
Global Assessment of Functioning (GAF) scale (1–100)	326	30 (15–50)	191	25 (10–40)	135	40 (20–50)	** < 0.001**	89	50 (40–60)	** < 0.001**
**Laboratory data in post-covid syndrome**										
IL-6 (pg/ml)	436	2.5 (2.5–3.4)	255	2.7 (2.5–3.7)	181	2.5 (2.5–2.9)	** < 0.001**	0	-	-
C-reactive protein (mg/l)	556	1.7 (0.8–4.2)	318	2.0 (1.0–4.6)	238	1.3 (0.6–3.5)	** < 0.001**	102	1.2 (0.6–2.5)	**0.005**
Ferritin (μg/l)	554	117.1 (67.2–201.9)	317	136.2 (81.7–237.6)	237	93.5 (49.2–170.4)	** < 0.001**	102	144.7 (76.1–274.3)	**0.033**
LDH (μmol/l*s)	510	3.3 (2.9–3.7)	285	3.5 (3.1–3.8)	225	3.1 (2.8–3.5)	** < 0.001**	99	3.5 (3.2–4.0)	** < 0.001**
AST (μmol/l*s)	403	0.37 (0.32–0.45)	233	0.39 (0.33–0.47)	170	0.36 (0.31–0.42)	**0.004**	100	0.42 (0.35–0.48)	**0.005**
ALT (μmol/l*s)	553	0.35 (0.28–0.52)	316	0.41 (0.29–0.62)	237	0.32 (0.25–0.41)	** < 0.001**	103	0.40 (0.30–0.52)	0.257
GGT (μmol/l*s)	554	0.31 (0.22–0.53)	316	0.40 (0.25–0.66)	238	0.26 (0.19–0.36)	** < 0.001**	103	0.36 (0.24–0.53)	0.105
Bilirubin (μmol/l)	554	7 (5–9)	317	6 (5–9)	237	7 (5–10)	0.082	103	6 (4–9)	0.066
Creatinine (μmol/l)	553	74 (66–83)	317	77 (67–87)	236	71 (65–80)	** < 0.001**	103	77 (66–87)	0.195
Leukocytes (Gpt/l)	556	6.5 (5.5–7.9)	318	6.8 (5.8–8.2)	238	6.2 (5.0–7.3)	** < 0.001**	103	6.9 (5.9–8.0)	**0.047**
Lymphocytes (Gpt/l)	547	1.8 (1.5–2.3)	312	2.0 (1.6–2.4)	235	1.8 (1.4–2.1)	** < 0.001**	98	2.1 (1.9–2.5)	** < 0.001**
INR	552	1.0 (0.9–1.0)	316	1.0 (0.9–1.0)	236	1.0 (1.0–1.0)	**0.007**	102	1.0 (1.0–1.1)	** < 0.001**
D-Dimer (μg/l)	552	95 (66–142)	315	99 (68–152)	237	90 (63–124)	**0.016**	0	-	-
Calprotectin (μg/g in stool)	474	25.3 (20–52.1)	269	26.7 (20.0–52.7)	205	23.7 (20.0–50.3)	0.213	0	-	-
SARS-CoV-2-IgG (AU/ml)	497	400 (58–1590)	285	353 (93–1540)	212	400 (39–1610)	0.675	0	-	-
FIB-4	402	0.91 (0.69–1.21)	233	0.96 (0.73–1.27)	169	0.82 (0.61–1.14)	**0.004**	100	1.13 (0.85–1.39)	** < 0.001**

Compared to 103 healthy controls (cohort 2), the PCS patients were slightly younger (50 vs 55 years, p < 0.001) and more frequently had psychiatric disorders (14.5 vs 6.9%, p = 0.039), while sex, BMI and other comorbidities did not differ between the groups. Notably, 90 of the control participants (87.4%) had a history of confirmed SARS-CoV-2 infection, which was more frequently non-severe compared to PCS patients (97.7 vs. 78.6%, p = 0.001). Only 2 control participants required hospitalization and oxygen support (2.2%). None was treated on an ICU compared to 7.0% of the PCS patients (p < 0.01) **(**Table 1**).**

As expected, PCS patients had a lower GAF score (60 vs 90, p < 0.001) and suffered from ongoing symptoms more frequently, with the exceptions of fever and abdominal pain. PCS patients had higher scores on the FAS (33 vs 22 points, p < 0.001) and BFI (5.4 vs 2.1, p < 0.001) indicating a higher level of fatigue. The average PHQ-9 was 11 in PCS patients and 3 in controls (p < 0.001), which can hint to depression but could also be a result of the fatigue. The PCS group achieved lower MoCA scores than the control group (27 vs 29 points, p < 0.001). (Table 1**).**

### Steatotic liver diseases in PCS (cohort 1)

Among the 560 PCS patients, 321 (57.3%) had signs of hepatic steatosis in ultrasound and TE, while 239 patients (42.3%) did not suffer from a steatotic liver disease (SLD). Within the control group, a similar proportion of 53.4% of patients had an SLD, which was not different from the PCS group (p = 0.516) **(**Fig. 2A** + B)**. Additionally, the severity of SLD as defined by CAP measurement did not differ between PCS patients and controls. The majority of patients with SLD had advanced (S3) steatosis in both groups (220 patients/39.3% in the PCS group and 33 patients/32% in the control group). 49 patients (8.8%) in the PCS group and 15 patients (14.6%) in the control group had signs of hepatic significant fibrosis in LSM (p = 0.071), of whom 8 patients (1.4%) in the PCS group and 3 patients (2.9%) in the control group suffered from advanced fibrosis/cirrhosis (LSM > 15 kPa). **(**Table 2**, **Fig. 2B**).**

**Table 2:** Steatotic liver diseases (SLD) and fibrosis in post-COVID-19 syndrome and healthy controls based on liver transient elastography (FibroScan®). *P* values from Mann-Whitney or Chi-squared test (bold value indicates statistical significance)

		Post-COVID-19 syndrome (n = 560)		Healthy controls (n = 103)	*P* value
CAP (db/m)	560	260 (219–306)	103	252 (219–302)	0.430
Steatosis grade S0 (CAP < 248 db/m) S1 (CAP 248–267 db/m) S2 (CAP 268–279 db/m) S3 (CAP ≥ 280 db/m)	560	239 (42.7) 65 (11.6) 36 (6.4) 220 (39.3)	103	48 (46.6) 13 (12.6) 9 (8.7) 33 (32.0)	0.478
LSM (kPa)	560	4.2 (3.5–5.5)	103	5.3 (4.1–6.4)	** < 0.0001**
Fibrosis grade F0/1 (LSM ≤ 7 kPa) F2 (LSM 7.1–9.9 kPa) F3 (LSM ≥ 10 kPa) F4 (LSM ≥ 13 kPa)	402	511 (91.2) 28 (5.0) 12 (2.1) 9 (1.6)	103	88 (85.4) 10 (9.7) 2 (1.9) 3 (2.9)	0.157

**Fig. 3:**
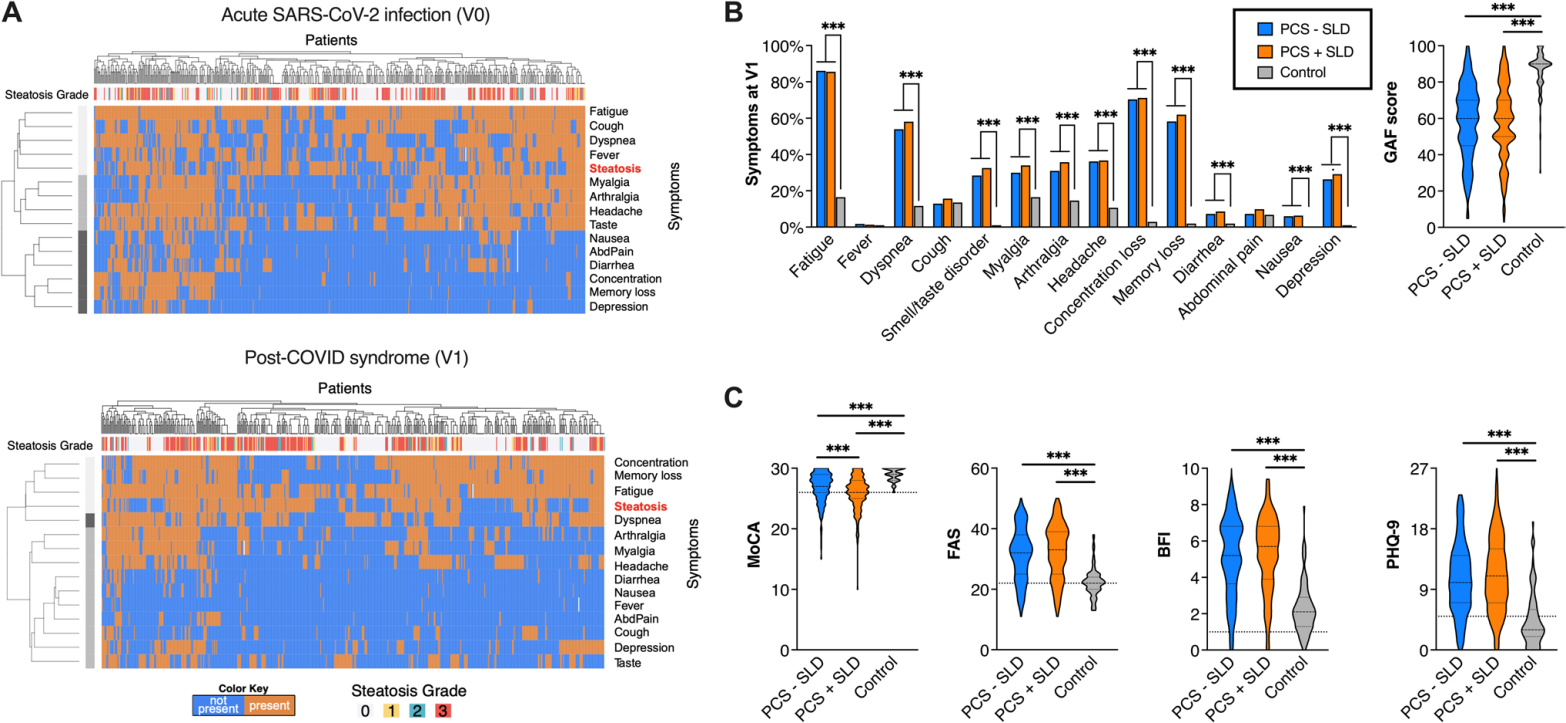
Complaints of post-COVID-19 syndrome (PCS) depending on steatotic liver diseases (SLD). **A** Cluster headmaps showing symptom complex depending on steatosis for acute SARS-CoV-2 infection (V0) and post-COVID condition (V1). **B** Symptom patterns at first clinical visit (V1) comparing patients with and without SLD. Global Assessment of Functioning (GAF) scale (0–100) was recorded at first presentation showing no differences between SLD and no SLD patients but compared to healthy controls. *P* value from Mann––Whitney U-test. **C** Neurocognitive screening in PCS patients (N = 560) and healthy controls (N = 103) determined by MoCA for cognitive dysfunction, FAS and BFI for fatigue und PHQ-9 for depression. PCS patients had the lowest MoCA scores at first presentation compared the no PCS and healthy controls. No differences were found in the PCS groups in term of fatigue and depression.* P* value from Mann––Whitney U-test

PCS patients with steatosis were more frequently male (41.4 vs. 24.3%, p < 0.001), older (52 vs. 44 years, p < 0.001) and had a higher BMI (29.7 vs. 23.8 kg/m^2^, p < 0.001) compared to PCS patients without steatosis. Cardiometabolic risk factors were more frequent in PCS patients with SLD than in those without (87.0 vs. 46.4%) (p < 0.001) **(**Fig. 2D**).** Regarding the acute course of the SARS-CoV-2 infection, patients with SLD had a critical outcome in 11.2% compared to 1.7% in patients without SLD (p < 0.001), which resulted in higher proportion of patients requiring oxygen (10.6 vs. 5%), NIV or HFNC (5.6 vs 0.4%) and invasive ventilation or ECMO (5.6 vs 1.3%) (p < 0.001). In line with this, the cluster analysis revealed that in acute SARS-CoV-2 infection, respiratory symptoms (cough, dyspnea, fever) were associated with the presence of SLD, whereas neurocognitive symptoms (concentration and memory loss, fatigue) tend to predominate in PCS with SLD. **(**Table 1**, **Fig. 3A** + B).**

PCS patients with and without SLD did not differ with respect to fatigue and functional status ratings (FAS 33 vs. 32 points, BFI 5.7 vs. 5.2 points, PHQ-9 11 vs. 10 points, all p > 0.05). Interestingly, the MoCA score, an index for neurocognitive dysfunction, was lower in PCS patients with steatosis than those without (26 vs. 27 points, p < 0.001) **(**Fig. 3C**).** This observation seemed to be independent of the primary disease severity of SARS-CoV-2 infection. This was also in line with outpatient cases with a non-severe course, where a higher proportion of neurocognitive dysfunction (measured by lower average MoCA scores) was observed in patients with SLD compared to those without steatosis (MoCA 26 vs. 27, p = 0.01). **(**Table 3**)**.

**Table 3:** Neurocognitive dysfunction in PCS patients with SARS-CoV-2 infection in an outpatient setting and depending on the presence of steatotic liver disease (SLD). *P* values from Mann-Whitney test (bold value indicates statistical significance)

		Outpatient SARS-CoV-2 infection without SLD (n = 211)		Outpatient SARS-CoV-2 infection with SLD (n = 229)	*P* value
MoCA	185	27 (26–29)	196	26 (25–28)	**0.010**
FAS	210	32 (25–38)	224	34 (27–39)	0.091
BFI	210	5.2 (3.7–6.8)	225	5.7 (4.2–6.8)	0.145
PHQ-9	210	10 (7–14)	225	11 (8–16)	0.156

Looking at routine laboratory parameters, the measured values were all within the normal range in median. Noteworthy, within the normal range, PCS patients with steatosis had slightly but significantly higher values of ALT (0.41 vs. 0.32 µkat, p < 0.001), AST (0.39 vs. 0.36 µkat, p = 0.004), GGT (0.40 vs. 0.26 µkat, p < 0.001) as well as CRP (2.0 vs. 1.3 mg/l, p < 0.001), IL-6 (2.7 vs. 2.5 pg/ml, p < 0.001), leukocytes (6.8 vs. 6.2 Gpt/l, p < 0.001), LDH (3.5 vs. 3.1 µkat, p < 0.001) and ferritin (136.2 vs. 93.5 µg/l, p < 0,001). These findings may indicate to a slightly pronounced hepatic inflammation in PCS patients, while fecal calprotectin as a marker of gastrointestinal inflammation did not differ between patients with and without steatosis (26.7 vs. 23.7 µg/g). The Fibrosis 4-Index (FIB-4) as surrogate marker for liver and cardiovascular events was significant enhanced in the PCS group with SLD compared to without chronic liver disease (0.96 vs. 0.82; p = 0.004). **(**Table 1**).**

### Risk factors associated with steatotic liver disease (SLD) in a joint sample (cohort 1 and 2)

**Fig. 4:**
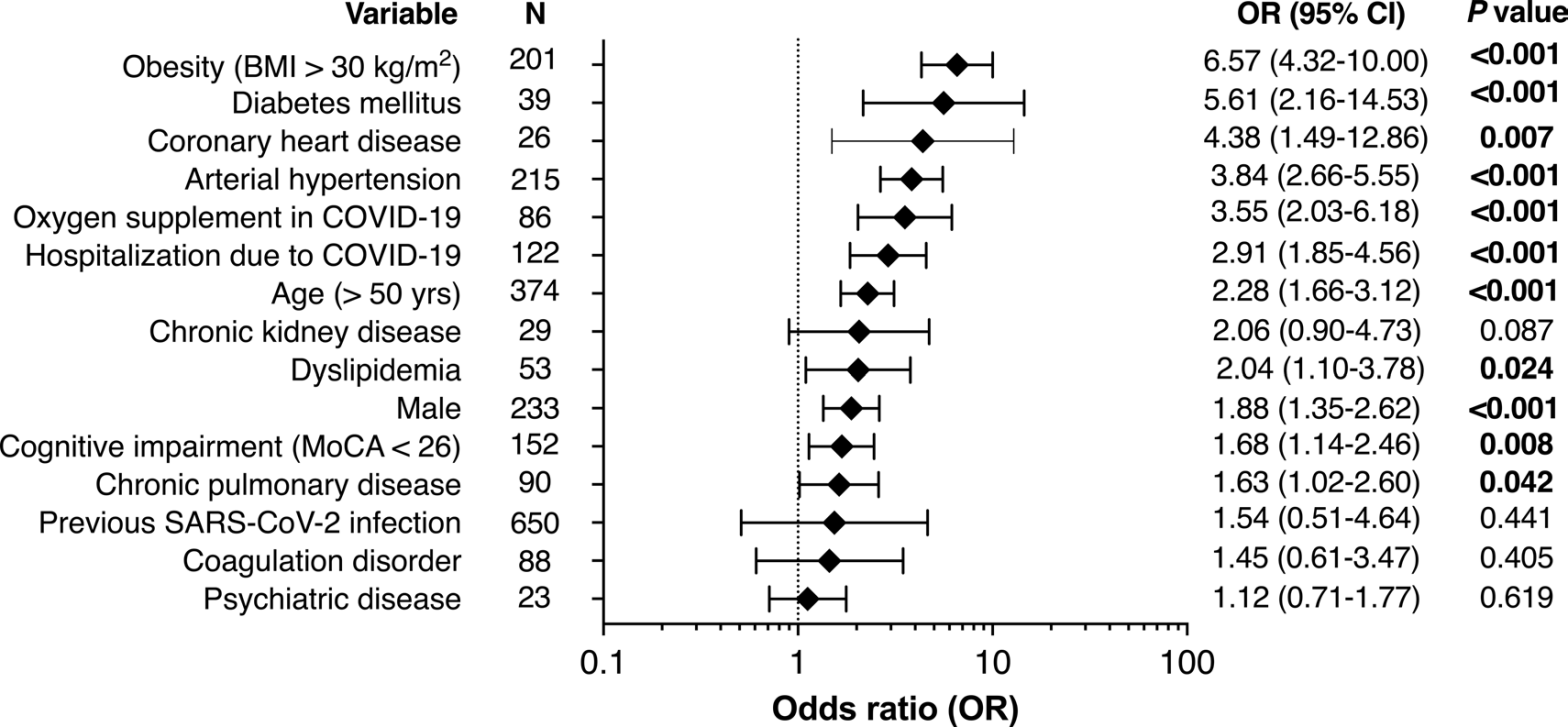
Risk factors associated with steatotic liver diseases (SLD) in total cohort. Forest plot of univariate logistic regression is shown for different subgroups based on odds ratios (OR) with 95% confidence interval. BMI, body-mass index. MoCa, Montreal Cognitive Assessement

To assess risk factors associated with SLD in the cohort we combined cohort 1 and 2 in one analysis. Risk factors for SLD included sociodemographic factors, i.e. age above 50 years (OR 2.28, 95%-CI 1.66–3.12), male sex (OR 1.88, 95%-CI 1.35–2.62), and comorbidities (BMI > 30 kg/m^2^ OR 6.57, 95%-CI 4.32–10.00; arterial hypertension OR 3.84, 95%-CI 2.66–5.55; coronary heart disease OR 4.38, 95%-CI 1.49–12.86; diabetes mellitus OR 5.61, 95%-CI 2.16–14.53; dyslipidemia OR 2.04, 95%-CI 1.10–3.78 and chronic pulmonary diseases OR 1.63, 95%-CI 1.02–2.60). A previous SARS-CoV-2 infection was not suggestive of SLD (OR 1.54, 95%-CI 0.51–4.64). However, a severe infection requiring hospitalization (OR 2.91, 95%-CI 1.85–4.56) or oxygen supply (OR 3.55, 95%-CI 2.03–6.18) was associated with SLD. Additionally, a MoCA below 26 points was linked to SLD (OR 1.68, 95%-CI 1.14–2.46). **(**Fig. 4**).**

### Course of PCS (cohort 1)

**Fig. 5:**
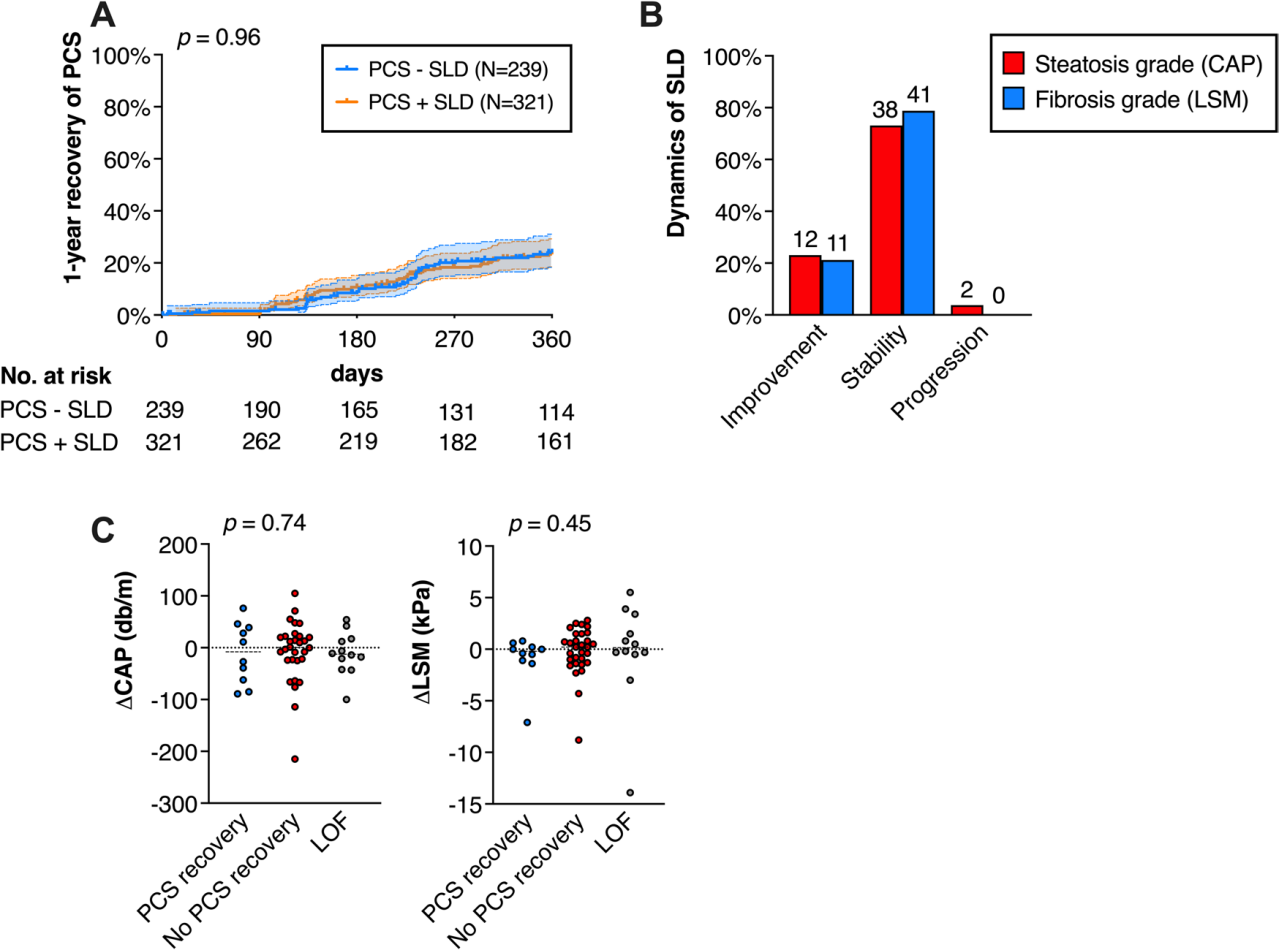
Clinical course of steatotic liver disease (SLD) in Post-COVID-19 syndrome (PCS). **A** 1-year recovery (with 95%-CI) of PCS in patients with SLD (N = 321) and without SLD (N = 239). Patients lost to follow-up (LOF) are censored. *P* value from Log-rank test. **B** Dynamic changes in PCS patients with SLD (n = 52) based on liver steatosis and stiffness between baseline and follow-up elastography measurements. Absolut values and frequencies are displayed. **C** Impact of differences in liver steatosis (ΔCAP) and stiffness (ΔLSM) on 1-year recovery of PCS.* P* value from Kruskal Wallis test. LOF, loss of follow-up

A total of 289 patients had a second visit in the post-COVID outpatient clinic (median 377 days after infection) and 97 patients had a third visit (median 515 days after infection). Within 1 year after the initial presentation 152 patients recovered from PCS, which was not different in patients with (88 patients/27.4%) and without (64 patients/26.8%) steatosis (log-rank test, p = 0.96) **(**Fig. 5A**).**

In this study, SLD was also not an independent predictor of PCS recovery within one year in Cox regression analysis. By contrast, cognitive deficits and severe fatigue (BFI ≥ 7) at initial presentation indicated a long-lasting course of PCS. (**supplemental **Table S1).

52 of the patients with SLD underwent repeated TE. Within this group, 12 patients (23.1%) improved in steatosis grade and 11 patients (21.2%) in fibrosis, while only 2 patients (3.8%) had a worsening steatosis grade (Fig. 5B**)**. However, the overall changes in CAP (p = 0.74) and LSM (p = 0.45) did not correlate with PCS recovery after one year **(**Fig. 5C**).** Neurocognitive screening did not reveal significant changes between the different visits in patients with PCS with/without SLD for the MoCA and the FAS score. Meanwhile, the BFI and the PHQ-9 showed variations without a clear trend, although these variations reached statistical significance (**supplemental **Figure S1**).**

## Discussion

In the present study we identified SLD as a frequent comorbidity in patients with PCS (57.3%) in one of the largest PCS cohorts of 560 patients. Furthermore, SLD was associated with lower cognitive functioning, as assessed by the MoCA, and with severe COVID-19 courses.

To date, the pathophysiology underlying PCS remains unclear [11]. Current hypotheses include persistence of viral epitopes, development of auto-immunity, including G-protein coupled autoantibodies [48], and vascular or endothelial damage [11], as well as metabolic changes [49]. Interestingly, comorbidities have also been described as a potential risk factor for PCS, particularly psychiatric disorders [50].

In our cohort, the prevalence of SLD was surprisingly high in both cohorts, patients with PCS as well as controls, with more than 50% affected in each cohort. The global prevalence of SLD varies depending on the region or cohort, but is currently considered to be 42–44% [20–22] with an increasing proportion of MASLD over the last decades, reaching up to 70% in the overweight population [51]. However, in line with current large cohort studies from Germany, the vast majority of patients in our cohort did not suffer from advanced liver fibrosis [52]. Compared to other PCS cohorts, the prevalence of hepatic steatosis in our cohort was nearly identical to that described by Milic et al. [13] following hospital discharge (57.3% vs. 55.3%). Milic et al. also estimated that approximately 37.3% of patients would have suffered from steatosis at hospital stay due to acute COVID-19.

In line with previous studies reporting an increased risk of a severe acute course of SARS-CoV-2 in patients with chronic liver diseases [16], our PCS patients with SLD were more frequently hospitalized and had a higher need for oxygen supply or respiratory support. Additionally, cluster analysis in our cohort revealed that respiratory symptoms in the acute phase were associated with SLD. In contrast, among PCS symptoms, neurocognitive dysfunction was associated with SLD, whereas persistent respiratory symptoms did not.

SLD is an already established risk factor for severe acute course [53], especially in the context of MASLD (reviewed by Nowroozi et al. [54]). In line with that our PCS patients with SLD had a more severe acute course of the SARS-CoV-2 infection as indicated by need for hospitalization (28.7 vs. 11.7%). Interestingly, in our control cohort, only 2.2% of participants required hospitalization due to COVID-19, although the prevalence of steatosis was over 50%.

It is possible that the suspected hepatotropism of SARS-CoV-2, along with consecutive inflammatory and metabolic changes plus genetic predisposition, can contribute to the development and the progression of liver steatosis in patients with pre-existing chronic liver disease [55]. A deep phenotypic characterization of PCS patients in terms of (auto)inflammation, metabolome, transcriptome and genetic variants could improve our understanding of the pathomechanism of PCS [56, 57] and the involvement of the liver. However, in accordance with our study protocol, we did not analyze any MALSD-associated genetic variants such as *PNPLA3*, *TM6SF2, GPAM/GPAT1 or APOE* [58] in our cohort. A review by Buchynskyi & Oksenych et al. also found no clear link between these risk variants and COVID-19 outcomes [59].

Our study has some limitations that must be acknowledged. First, due to the cross-sectional study design, we lack data on the presence of SLD prior to the SARS-CoV-2 infection. Therefore, we cannot differentiate for sure whether the reported symptoms are really “post-COVID symptoms”, since these strict definitions require “symptoms that cannot be explained otherwise” [3]. Since chronic liver disease can also cause fatigue and cognitive disorders, it could be argued that patients with pre-existing SLD do not suffer from PCS, as their symptoms can be explained by SLD. However, in clinical practice, this strict definition is not always helpful. Patients seek medical attention for specific symptoms [60] and therefore, if they suffer from SLD and potential PCS, both will be addressed as therapeutic targets. Furthermore, the inclusion of PCS patients with a broad range of COVID-19 severities (ranging from non-hospitalized individuals to ICU admission) led to heterogeneity complicating the analyses.

Second, we did not collect data on daily alcohol consumption for all patients in our cohort in accordance with the newly established concept of the SLD subclassification [23]. Data on self-reported alcohol intake is often subject to concern, as many patients underreport their alcohol consumption. Therefore, as the recent MASLD-concept allows alcohol intake also in patients suffering from metabolic liver disease, we decided against asking for this potentially confounded data. Furthermore, not all PCS patients were excluded from further etiologies (viral, autoimmune) of SLD. Third, selection bias is likely in the recruitment of our control cohort. Although none of the participants had chronic liver disease, some exhibited critical alcohol consumption and took advantage of the liver screening.

Our study suggests a link between SLD and cognitive impairment in PCS but does not establish causality. Longitudinal follow-up or mechanistic studies would be needed to differentiate whether liver dysfunction contributes to neurocognitive symptoms or is a parallel phenomenon.

## Conclusions

SLD is common in PCS with a prevalence of almost 60%. It is associated with neurocognitive impairment, severe COVID-19 cases, older age, male sex und cardiometabolic risk factors. Severe fatigue and cognitive dysfunction, but not SLD, are predictors of the course of PCS. Due to limitations of the study, no clear recommendation can be offered regarding screening or management of SLD in patients with PCS.

Medians with interquartile range and frequencies are given. *P* values are calculated with Mann––Whitney U-test or Fisher’s exact test. *ALAT* alanine aminotransferase, *ASAT* aspartate aminotransferase, *BMI* body-mass index, *ECMO*, extracorporeal membrane oxygenation, *FIB-4* Fibrosis-4 score. GAF, Global Assessment of Functioning. GGT, gamma-glutamyl transferase. HFNC, high-flow nasal cannula. LDH, lactate dehydrogenase. INR, International Normalized Ratio. NIV, noninvasive mechanical ventilation.

Absolut and relative values are calculated. *P* values are calculated with Fisher’s Exact and Chi-Square test. CAP, controlled attenuation parameter. LSM, liver stiffness measurement. SLD, steatotic liver disease.

Medians with interquartile range and frequencies are given. *P* values are calculated with Mann–Whitney U-test. BFI, Brief Fatigue Inventory, *FAS* Fatigue Assessment Scale, *MoCA* Montreal Cognitive Assessment, *PHQ-9* Patient Health Questionnaire, *SLD* steatotic liver disease.

## Supplementary Information

Below is the link to the electronic supplementary material.

Supplementary file1 (DOCX 1029 KB)

## Data Availability

No datasets were generated or analysed during the current study. The data underlying this manuscript are available from the corresponding author upon reasonable request.

## Abbreviations

ALAT: Alanine aminotransferase.
ASAT: Aspartate aminotransferase.
BFI: Brief Fatigue Inventory.
BMI: Body-mass index.
CAP: Controlled attenuation parameter.
COVID: Corona-Virus-Disease.
ECMO: Extracorporeal membrane oxygenation.
FAS: Fatigue Assessment Scale.
GAF score: Global Assessment of Functioning score.
GGT: Gamma-glutamyl transferase.
HFNC: High-flow nasal cannula.
LDH: Lactate dehydrogenase.
LSM: Liver stiffness measurement.
MASLD: Metabolic dysfunction associated steatotic liver disease.
MoCA: Montreal Cognitive Assessment.
NAFLD: Non-alcoholic fatty liver disease.
NIV: Noninvasive mechanical ventilation.
PCS: Post-COVID syndrome.
PHQ-9: Patient Health Questionnaire.
SARS-CoV-2: Severe acute respiratory syndrome coronavirus 2.
SLD: Steatotic liver disease.
TE: Transient elastography.
