# Supplementary material for: Possible link between steatotic liver diseases, severe COVID-19 and cognitive impairment in post-COVID-19 syndrome
Source: Infection. 2025 Apr 10;53(5):1979–92. doi: 10.1007/s15010-025-02531-x (PMC12460423; doi:10.1007/s15010-025-02531-x)
Supplement: Supplementary file 1 — Supplementary file1 (DOCX 1029 KB) [file 15010_2025_2531_MOESM1_ESM.docx]

**SUPPLEMENT**

**Table S1. Predictors of PCS recovery in univariate and multivariate models using Cox regression analysis.**

|  | **Univariate analysis** | | **Multivariate analysis** | |
| --- | --- | --- | --- | --- |
| **Variable** | **Hazard with 95% CI** | ***P* value** | **Hazard with 95% CI** | ***P* value** |
| SLD | 0.990 (0.663-1.477) | n.s. | 1.093 (0.645-1.855) | n.s. |
| Age > 50 yrs | 1.017 (0.682-1.516) | n.s. | 1.011 (0.641-1.594) | n.s. |
| Male | 1.149 (0.758-1.741) | n.s. | 1.301 (0.823-2.057) | n.s. |
| Cardiometabolic risk factors | 1.055 (0.674-1.653) | n.s. | 1.005 (0.553-1.825) | n.s. |
| Obesity (BMI > 30 kg/m^2^) | 1.093 (0.719-1.662) | n.s. | 1.160 (0.690-1.951) | n.s. |
| Coagulation disorder | 0.678 (0.215-2.142) | n.s. | - | - |
| Psychiatric disease | 1.099 (0.643-1.878) | n.s. | 1.182 (0.659-2.117) | n.s. |
| Hospitalization due to COVID-19 | 1.203 (0.754-1.919) | n.s. | - | - |
| Oxygen supplement in COVID-19 | 1.069 (0.617-1.852) | n.s. | - | - |
| Cognitive impairment (MoCA < 26) | 0.465 (0.269-0.803) | **0.006** | 0.484 (0.278-0.842) | **0.01** |
| Severe Fatigue (BFI ≥ 7) | 0.226 (0.105-0.487) | **<0.001** | 0.245 (0.106-0.566) | **<0.001** |

**Figure S1. Neurocognitive screening over time with two further visits (V2 and V3) in PCS patients**. *P* values were calculated with Mann-Whitney U-test and Wilcoxon test. BFI, Brief Fatigue Inventory. FAS, Fatigue Assessment Scale. MoCA, Montreal Cognitive Assessment. PCS, Post-COVID syndrome. PHQ-9, Patient Health Questionnaire. SLD, steatotic liver disease.
